# Identifying risk profiles for childhood obesity using recursive partitioning based on individual, familial, and neighborhood environment factors

**DOI:** 10.1186/s12966-015-0175-7

**Published:** 2015-02-15

**Authors:** Andraea Van Hulst, Marie-Hélène Roy-Gagnon, Lise Gauvin, Yan Kestens, Mélanie Henderson, Tracie A Barnett

**Affiliations:** Département de médecine sociale et préventive, École de santé publique de l’Université de Montréal, Montreal, Quebec Canada; Centre de recherche du Centre Hospitalier Universitaire Sainte-Justine, 5757 Avenue Decelles, suite 100, Montréal, H3S 2C3 Québec Canada; Department of Epidemiology and Community Medicine, Faculty of Medicine, University of Ottawa, Ottawa, Ontario Canada; Centre de Recherche du Centre Hospitalier de l’Université de Montréal (CRCHUM), Montreal, Quebec Canada; Centre de Recherche Léa-Roback sur les Inégalités Sociales de Santé de Montréal, Université de Montréal, Montreal, Quebec Canada; Division of Endocrinology, Department of Pediatrics, Centre Hospitalier Universitaire Sainte-Justine and Université de Montréal, Montreal, Quebec Canada; Epidemiology and Biostatistics Unit, INRS-Institut Armand-Frappier, Université du Québec, Laval, QC Canada

**Keywords:** Built environment, Body mass index, Familial risk, Food environment, Neighborhood characteristics, Obesity, Physical activity, Recursive partitioning analysis, Socioecological model

## Abstract

**Background:**

Few studies consider how risk factors within multiple levels of influence operate synergistically to determine childhood obesity. We used recursive partitioning analysis to identify unique combinations of individual, familial, and neighborhood factors that best predict obesity in children, and tested whether these predict 2-year changes in body mass index (BMI).

**Methods:**

Data were collected in 2005–2008 and in 2008–2011 for 512 Quebec youth (8–10 years at baseline) with a history of parental obesity (QUALITY study). CDC age- and sex-specific BMI percentiles were computed and children were considered obese if their BMI was ≥95^th^ percentile. Individual (physical activity and sugar-sweetened beverage intake), familial (household socioeconomic status and measures of parental obesity including both BMI and waist circumference), and neighborhood (disadvantage, prestige, and presence of parks, convenience stores, and fast food restaurants) factors were examined. Recursive partitioning, a method that generates a classification tree predicting obesity based on combined exposure to a series of variables, was used. Associations between resulting varying risk group membership and BMI percentile at baseline and 2-year follow up were examined using linear regression.

**Results:**

Recursive partitioning yielded 7 subgroups with a prevalence of obesity equal to 8%, 11%, 26%, 28%, 41%, 60%, and 63%, respectively. The 2 highest risk subgroups comprised i) children not meeting physical activity guidelines, with at least one BMI-defined obese parent and 2 abdominally obese parents, living in disadvantaged neighborhoods without parks and, ii) children with these characteristics, except with access to ≥1 park and with access to ≥1 convenience store. Group membership was strongly associated with BMI at baseline, but did not systematically predict change in BMI.

**Conclusion:**

Findings support the notion that obesity is predicted by multiple factors in different settings and provide some indications of potentially obesogenic environments. Alternate group definitions as well as longer duration of follow up should be investigated to predict change in obesity.

**Electronic supplementary material:**

The online version of this article (doi:10.1186/s12966-015-0175-7) contains supplementary material, which is available to authorized users.

## Background

Childhood obesity has reached epidemic proportions worldwide [1] and its health consequences are considerable [2]. Obesity is a complex condition in which a myriad of risk factors interact within and between several levels of influence [3]. Social ecological frameworks posit that childhood obesity is influenced by energy intake and expenditure patterns, which are embedded within the familial and wider community contexts [4-6]. An understanding of the multiple influences on obesity, including within individual, familial, and neighborhood levels, will improve population efforts to address childhood obesity. For example, at the individual level, regular intake of sugar-sweetened beverages [7] and physical inactivity [8] have been associated with childhood obesity. Similarly, through shared genetics and lifestyles, parental obesity has been identified as a risk factor for childhood obesity [4,9,10]. Within wider community contexts, neighborhood parks, sports and recreational facilities, and the presence of nearby convenience stores and fast food restaurants have been associated with childhood obesity, albeit inconsistently [6,11-14]. Neighborhood disadvantage has been more consistently associated with childhood obesity [15,16]. However, it remains unclear how factors within these different levels of influence interact to determine obesity.

Individual, familial, and neighborhood factors may have synergistic effects on childhood obesity [17,18]. To test hypotheses regarding synergistic effects (i.e., effect modification), interaction terms in regression models are typically used [18]. However, this approach is not ideal for modeling more complicated nonlinear associations. An alternative non-parametric method consists of using recursive partitioning analysis, which has gained popularity as a means of multivariate data exploration in various fields [19]. Recursive partitioning produces a classification tree following a series of binary splits dividing children into higher- and lower-risk subgroups for a given outcome based on a number of predictor variables [20]. In addition to its intuitive appeal, recursive partitioning methods are particularly useful to examine higher order interactions, for example between multiple individual and neighborhood characteristics [21]. Therefore, the primary objective of this study is to determine optimal combinations of individual, familial, and neighborhood environment characteristics that best predict obesity among children using recursive partitioning analysis. A secondary objective is to examine whether the resulting classification is associated with 2-year changes in body mass index (BMI) percentile.

## Methods

### Subjects

Participants were drawn from QUALITY (Quebec Adipose and Lifestyle Investigation in Youth), an ongoing longitudinal investigation of the natural history of obesity and cardiovascular risk in Quebec youth. At baseline, 630 participants aged 8 to 10 years were recruited using school-based sampling (2005–2010). Eligibility criteria, verified over the phone, required participating children to have at least 1 obese biological parent based on parent-reported measurements of weight, height, and waist circumference (i.e., BMI ≥30 kg/m^2^ and/or waist circumference >102 cm in men and >88 cm in women). At the baseline clinic visit, parental anthropometrics were measured. Thirty-five children had no obese parents based on measured BMI or waist circumference, likely due to self-report measurement error or to weight loss between the initial contact and the baseline visit; these families were nevertheless retained since inclusion criteria were based a priori on self-report and since children still had at least 1 borderline obese parent. A 2-year follow-up assessment was completed in 2008–2011. Characteristics of neighborhood environments were assessed at baseline for participants residing in the Montreal Metropolitan Area (n = 512) to which this study is restricted. The ethics review boards of CHU Sainte-Justine and Laval University approved the study protocol. A detailed description of the study design and methods is available elsewhere [22].

### Measurement of individual characteristics

Child anthropometrics were measured at baseline and follow-up using standardized protocols [22]. Centers for Disease Control and Prevention age- and sex-specific BMI percentiles were computed. Children were categorized as obese if their BMI was ≥95^th^ percentile. Pubertal development stage was assessed by a nurse using the 5-stage Tanner scales [23,24], and was dichotomized as pre-pubertal (Tanner 1) Vs. puberty initiated (Tanner >1) for both baseline and follow-up.

Intake of sugar-sweetened beverages was measured using mean values of 3 24-hour diet recalls conducted by trained dieticians on non-consecutive days including 1 weekend day [25]. Except in unusual circumstances, the recalls were collected within a 4-week period following the baseline clinic visit. Diet recall interviews were done by telephone with the child and then confirmed with the parent who prepared the meals. Reported foods were entered into CANDAT (London, Canada) and converted to nutrients using the 2007b Canadian Nutrient File [26]. Intake of sugar-sweetened beverages was computed as the mean daily mL of soft drinks and other sugar-sweetened drinks, excluding juices made from real fruits. Given a substantial positive skewness in its distribution, the variable was dichotomised to >50 mL/day (approximately 1 soft drink can per week) Vs. less.

Participants’ physical activity (PA) was measured using a uniaxial activity monitor (Actigraph LS 7164 activity monitor, Actigraph) for 7 days during the week following the baseline clinic visit. A minimum of 4 days with ≥10 h of wear time was required for data to be retained [27]. The Actigraph cut-offs proposed by Evenson et al. were used to define moderate to vigorous PA (MVPA) [28]. Based on Canadian PA guidelines, children achieving a mean of at least 60 minutes of MVPA per valid day were classified as active.

### Measurement of familial characteristics

At baseline, parents’ weight, height, and waist circumference were measured using standardized protocols [22]. Two parental obesity variables were examined: BMI-defined obesity (BMI ≥30 kg/m^2^) and abdominal obesity (waist circumferences >88 cm for mothers and >102 cm for fathers) [29]. For both parental obesity variables, children were categorized as having none, 1 or 2 obese parents. Highest parental educational attainment and total annual household income adjusted for the number of people living in the household were obtained from parent-completed questionnaires during clinic visits.

### Measurement of neighborhood environment characteristics

Neighborhood environments were characterized using a geographic information system (GIS) for the study area. Canadian Census data from 2006 were used to obtain the following measures: % residents with a university degree, average value of owner occupied residences, % households living below Statistics Canada’s low income cut-offs [30], % single parent families, % unemployment, % who have moved in the past year and % owner occupied residences. For each measure, population-weighted proportions or averages of Census dissemination areas overlapping 500 m network buffers centered on the child’s residential address were computed. These variables were then reduced to 2 components using principal components analysis, namely neighborhood prestige (university degree and housing value) and neighborhood disadvantage (remaining Census variables described above), and then categorized into tertiles (see Additional file 1: Table S1) [31].

The GIS also provided information on food establishments located within 500 m network buffers around the residence based on data from an exhaustive list of businesses and services located in the region in May 2005 acquired from Tamec Inc. A validation study of food establishments from this list, verified by onsite field visits showed good agreement (0.77), sensitivity (0.84), and positive predictive value (0.90) [32]. All businesses were geocoded using DMTI GeoPinPoint, version 2007.3. In this study we focused on access to convenience stores and fast food restaurants based on evidence of associations with unhealthful diets [33]. Children were categorised as living within ≥1 convenience store (Vs. not) and within ≥1 fast food restaurant (Vs. not) located in 500 m network buffers centered on their residence given our hypothesis that having proximal access to any such amenity relative to none is sufficient to influence access.

Lastly, the presence of parks was computed using land use information from CanMap (DMTI Spatial Inc.). Information from GIS identified parks was subsequently validated by in-person neighborhood assessments during which independent pairs of trained observers walked every street within 500 m network buffers centered on participants’ residences. Parks were defined as public open spaces in which children could engage in active play. Participants were classified as having or not ≥1 park within 500 m network buffers centered on their residence. All neighborhood environment measurements were operationalized for 500 m network buffers given that children and youth typically have smaller activity spaces than adults and for the sake of consistency in buffer size given that observer-validated park counts were available only for 500 m network buffers.

### Statistical analysis

Recursive partitioning was used to identify subgroups of participants that varied in terms of obesity using the RPART routine available in the R statistical environment [34]. This non-parametric regression method produces a classification tree following a series of non-sequential top-down binary splits. The tree-building process starts by considering a set of predictor variables and selects the variable that produces 2 subsets of participants with the greatest purity (i.e., where participants within each subset are most alike in terms of the outcome). Two factors are considered when splitting a node into its daughter nodes: the goodness of the split and the amount of impurity in the daughter nodes [35]. The splitting process is repeated until further partitioning is no longer possible and terminal nodes have been reached. Because the resulting tree is typically large, difficult to interpret, and may over-fit to the data, pruning techniques are used to reduce the size of the original tree by eliminating selected branches from later splits. This is done using cost-complexity measures and cross-validations to assess the predictive performance of several reduced subtrees. The final classification tree is a subtree of the original tree that is most predictive of the outcome and has the lowest cross-validated error [19].

Observations that have missing values on a predictor variable are not discarded from the analysis. Instead, these observations are ignored for the computation of the impurity index when that variable is being considered as a splitting variable, but they are included in subsequent computations. To do so, a surrogate variable that best predicts the missing splitting values is used to determine the classification of observations with missing values to either daughter node (see Strobl et al. for details [19]).

**Figure 1:**
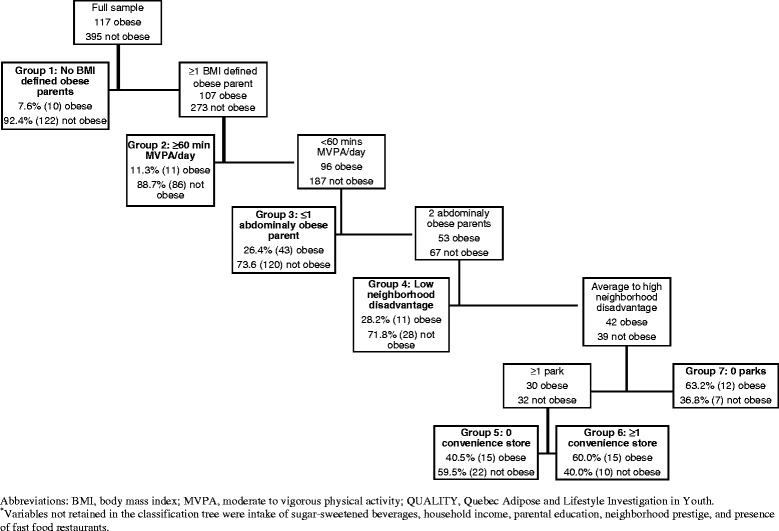
**Classification tree obtained from recursive partitioning analysis of individual, familial, and neighborhood factors** ^*^ **in 512 QUALITY study participants at baseline (2005-2008).**

In this study, 9 variables were submitted to the recursive partitioning process, based on evidence of associations with childhood obesity: 2 individual variables (sugar-sweetened beverage intake, meeting PA guidelines), 4 familial variables (number of BMI-defined obese parents, number of parents with abdominal obesity, parental education, household income), and 5 neighborhood environment characteristics (disadvantage, prestige, and presence of ≥1 park, fast food restaurant, and convenience store). The Gini index was used as an indicator of node purity which reaches its minimum for perfectly pure nodes (the desired result) and its maximum when cases are distributed evenly between classes at a given node [19]. A 10-fold cross-validation technique was used to prune the tree; the best tree was based on the “1 –SE” rule in which the cross-validated error estimate is no more than 1 standard error (SE) larger than the best tree [19,36]. This resulted in classification trees with 7 terminal nodes (Figure 1).

Multivariable linear regression models were subsequently used to examine associations between the categorical variable that represents the recursive partitioning subgroups (terminal nodes) and BMI percentile while controlling for age, sex, puberty, and parental education. The lowest risk subgroup was the reference category; the remaining subgroups were identified using 6 indicator variables. Finally, associations between subgroup membership and BMI percentile at follow-up were examined while adjusting for BMI percentile at baseline. These analyses were conducted with SAS version 9.3 (Cary, North-Carolina). Although a school-based sampling was used in QUALITY, clustering of participants in schools did not significantly influence estimates for associations (see Additional file 1: Tables S2 and S3).

## Results

Characteristics of study participants are provided in Table 1. Both at baseline and at follow-up, 23% of participants were obese (117/512 and 106/462, respectively). Thirty four percent of obese participants had initiated puberty at baseline compared to 21% among non-obese participants. At follow-up, 77% of obese and 66% of non-obese participants had initiated puberty. Overall, more than half consumed >50 mL of sugar-sweetened beverage per day and obese participants were less likely to engage in ≥60 minutes of MVPA daily. Familial characteristics varied according to obesity status in the expected direction with a greater proportion of obese children in lower income/education households, and in households with 2 obese parents (defined using BMI or waist circumference). Obese children more often lived in neighborhoods characterised by high disadvantage and by the proximity to ≥1 convenience stores.

**Table 1:** **Distribution of individual, familial, and neighbourhood characteristics according to obesity status (BMI ≥95** ^**th**^ **percentile) among QUALITY study participants at baseline in 2005-2008**

	**Obese**	**Not obese**	
	**(n = 117)**	**(n = 395)**	P value*
**Individual characteristics**			
Age, years, mean (sd)	9.7 (0.9)	9.6 (0.9)	0.11^*^
Sex, boys,% (n)	51.3 (60)	55.4 (219)	0.43
Puberty initiated at baseline,% (n)	33.6 (39)	21.0 (83)	0.005
Puberty initiated at follow-up,% (n)	76.8 (76)	65.8 (237)	0.04
Sugar-sweetened beverage intake >50 ml/day,% (n)^§^	65.8 (75)	55.7 (214)	0.06
Meet physical activity guidelines,% (n)^§§^	12.8 (12)	37.4 (127)	<0.001
BMI percentile at baseline, mean (sd)	97.8 (1.3)	60.3 (26.8)	<0.001^*^
BMI percentile at follow-up, mean (sd)	96.5 (5.1)	61.2 (27.3)	<0.001^*^
**Familial characteristics**			
Household income <25000$,% (n)	26.1 (30)	15.1 (59)	0.006
Highest level of education of either parent,% (n)			
2 parents with high school degree or less	14.7 (17)	6.4 (25)	<0.001
≥1 parent with technical/vocational/trade school degree	47.4 (55)	35.9 (141)	
≥1 parent with university degree	37.9 (44)	57.8 (227)	
Number of parents with BMI ≥30 kg/m^2^,% (n)			
0	8.6 (10)	30.9 (122)	<0.001
1	62.4 (73)	52.4 (207)	
2	29.1 (34)	16.7 (66)	
Number of parents with abdominal obesity,% (n)			
0	4.3 (5)	10.1 (40)	<0.001
1	44.4 (52)	61.8 (244)	
2	51.3 (60)	28.1 (111)	
Mother’s BMI, kg/m^2^, mean (sd)	31.9 (7.3)	28.8 (6.2)	<0.001^*^
Father’s BMI, kg/m^2^, mean (sd)	32.9 (6.2)	30.2 (5.3)	<0.001^*^
Mother’s waist circumference, cm, mean (sd)	99.6 (15.6)	92.0 (13.9)	<0.001^*^
Father’s waist circumference, cm, mean (sd)	111.4 (16.1)	105.2 (13.6)	<0.001^*^
**Neighborhood characteristics**			
% residents with a university degree, mean (sd)	26.6 (14.4)	29.1 (15.5)	0.12^*^
Residential value, $1000, mean (sd)	204 (52)	215 (61)	0.07^*^
Neighborhood prestige,% (n)			
Low	37.6 (44)	31.9 (126)	0.42
Average	33.3 (39)	33.4 (132)	
High	29.1 (34)	34.7 (137)	
% households with low income, mean (sd)	8.0 (6.6)	7.3 (6.7)	0.29^*^
% single parent families, mean (sd)	16.7 (7.0)	15.3 (7.1)	0.05^*^
% unemployment, mean (sd)	5.3 (2.6)	5.2 (3.0)	0.72^*^
% 1 year mobility, mean (sd)	11.0 (5.2)	10.8 (5.4)	0.63^*^
Home ownership, mean (sd)	67.8 (25.5)	74.5 (25.8)	0.08^*^
Neighborhood disadvantage,% (n)			
Low	25.6 (30)	35.4 (140)	0.09
Average	34.2 (40)	33.2 (131)	
High	40.2 (47)	31.4 (124)	
≥1 park within 500 m,% (n)	68.4 (80)	74.9 (296)	0.16
≥1 convenience store within 500 m,% (n)	35.9 (42)	26.8 (106)	0.06
≥1 fast food restaurant within 500 m,% (n)	17.1 (20)	11.9 (47)	0.14

*Abbreviations:* BMI, Body mass index; QUALITY, Quebec Adipose and Lifestyle Investigation in Youth; sd, standard deviation.

*The P value of a t-test comparing mean values between obese and non-obese.

^§^Data missing for 14 (3 obese and 11 non-obese) participants.

^§§^Data missing for 67 (19 obese and 48 non-obese) participants.

The classification tree showed sequentially increasing prevalence of obesity in its 7 terminal nodes (Figure 1). The lowest risk subgroup, Group 1 (i.e., reference), consisted of 132 participants with no BMI-defined obese parent (8% obese). Group 2 consisted of 97 participants with ≥1 BMI-defined obese parent but who meet PA guidelines (11% obese). Group 3 consisted of 163 participants with ≥1 BMI-defined obese parent, not meeting PA guidelines, and with ≤1 abdominally obese parent (26% obese). Group 4 consisted of 39 participants with ≥1 BMI-defined obese parent, not meeting PA guidelines, with 2 abdominally obese parents, and living in a low disadvantage neighborhood (28% obese). Group 5 consisted of 37 participants with ≥1 BMI-defined obese parent, not meeting PA guidelines, with 2 abdominally obese parents, living in an average to high disadvantage neighborhood with ≥1 park and no convenience store (41% obese). Group 6 consisted of 25 participants with ≥1 BMI-defined obese parent, not meeting PA guidelines, with 2 abdominally obese parents, living in an average to high disadvantage neighborhood with ≥1 park but also to ≥1 convenience store (60% obese). Lastly, Group 7 consisted of 19 participants with ≥1 BMI-defined obese parent, not meeting PA guidelines, with 2 abdominally obese parents, living in an average to high disadvantage neighborhood with no access to parks or to convenience stores (63% obese).

Recursive partitioning successfully generated subgroups that differed in obesity status. After adjusting for child’s age, sex, pubertal development stage, and parental education at baseline, children from Groups 2 to 7 had sequentially increasing BMI percentiles, varying from 12 [Group 2, B = 12.3 (95% confidence interval (CI): 5.3; 19.3)] to 33 [Group 7, B = 32.7 (95% CI: 19.9; 45.4)] percentile points higher compared to children with no BMI-defined obese parent (Group 1) (Table 2).

**Table 2:** **Unadjusted and adjusted associations (beta coefficients and 95% CIs) between risk subgroups identified using recursive partitioning analysis and body mass index percentile among 512 QUALITY study participants at baseline (2005–2008)**

	**Beta (95% CI)**	
Intercept	54.9 (50.4; 59.5)	78.4 (51.9; 105.0)
Group 1 (n = 132), obesity prevalence 7.6%	Reference	Reference
Group 2 (n = 97), obesity prevalence 11.3%	13.3 (6.3; 20.3)	12.3 (5.3; 19.3)
Group 3 (n = 163), obesity prevalence 26.4%	16.0 (9.8; 22.1)	15.8 (9.6; 22.0)
Group 4 (n = 39), obesity prevalence 28.2%	22.0 (12.4; 31.6)	22.6 (13.1; 32.1)
Group 5 (n = 37), obesity prevalence 40.5%	25.1 (15.3; 34.8)	23.8 (14.1; 33.5)
Group 6 (n = 25), obesity prevalence 60.0%	31.9 (20.4; 43.3)	31.8 (20.4; 43.1)
Group 7 (n = 19), obesity prevalence 63.2%	34.4 (21.6; 47.3)	32.7 (19.9; 45.4)
Child’s age		−3.3 (−6.1; −0.5)
Sex, boys (Vs. girls)		6.7 (1.5; 11.8)
Puberty initiated at baseline (Vs. not initiated)		10.4 (3.9; 16.9)
Parental education		
≥1 parent with university degree		Reference
≥1 parent with technical/vocational/trade school degree		5.0 (0.1; 9.9)
2 parents with high school degree or less		7.6 (−1.1; 16.2)

*Abbreviations:* CI, Confidence interval; QUALITY, Quebec Adipose and Lifestyle Investigation in Youth.

Follow-up data were available for 462 participants. Of the 50 participants lost to follow-up, almost half (46% n = 23) were lost from Group 3, of which 39% (n = 9) were obese at baseline. Changes in BMI percentile between baseline and follow-up are shown in Tables 3 and 4. Only Group 3 (≥1 BMI-defined obese parent, not meeting PA guidelines, and with ≤1 abdominally obese parent) showed an increase in BMI percentile after a 2-year follow-up in comparison to Group 1 [B = 3.6 (95% CI: 0.5; 6.6)] (Table 3).

**Table 3:** **Unadjusted and adjusted associations (beta coefficients and 95% CIs) between risk subgroups identified using recursive partitioning analysis and body mass index percentile among 462 QUALITY study participants at 2 year follow-up (2005–2011)**

	**Beta (95% CI)**	
Intercept	5.1 (1.9; 8.3)	1.8 (−13.9; 17.5)
Child’s BMI percentile at baseline	0.9 (0.9; 0.9)	0.90 (0.9; 0.9)
Group 1 (n = 123), obesity prevalence 8.9%	Reference	Reference
Group 2 (n = 88), obesity prevalence 13.6%	1.4 (−2.0; 4.7)	1.5 (−1.8; 4.9)
Group 3 (n = 140), obesity prevalence 27.1%	3.8 (0.8; 6.8)	3.6 (0.5; 6.6)
Group 4 (n = 37), obesity prevalence 16.2%	−0.1 (−4.6; 4.5)	−0.1 (−4.7; 4.4)
Group 5 (n = 34), obesity prevalence 35.3%	3.7 (−1.0; 8.4)	3.8 (−1.0; 8.6)
Group 6 (n = 23), obesity prevalence 65.2%	1.1 (−4.5; 6.6)	1.0 (−4.6; 6.6)
Group 7 (n = 17), obesity prevalence 70.6%	2.9 (−3.4; 9.2)	2.5 (−3.8; 8.8)
Child’s age at follow-up, years		0.1 (−1.3; 1.5)
Sex, boys (Vs. girls)		0.7 (−1.8; 3.1)
Puberty initiated at follow-up (Vs. not initiated)		2.6 (−0.3; 5.5)
Parental education		
≥1 parent with university degree (reference)		Reference
≥1 parent with technical/vocational/trade school degree		−0.03 (−2.4; 2.3)
2 parents with high school degree or less		0.5 (−3.8; 4.8)

*Abbreviations:* BMI, Body mass index; CI, Confidence interval; QUALITY, Quebec Adipose and Lifestyle Investigation in Youth.

**Table 4:** **Distribution of obesity at baseline and 2-year follow-up according to subgroups identified using recursive partitioning analysis among 462 QUALITY study participants at 2 year follow-up (2005–2011)**

**Recursive partitioning subgroups**	**Obese at baseline**	**Obese at follow-up**	**BMI % at baseline**	**BMI % at follow-up**	**Change in BMI %**
(n)	% (n)		Mean (sd)		
Group 1 (123)	7.3 (9)	8.9 (11)	56.1 (28.2)	55.8 (29.0)	−0.3 (14.2)
Group 2 (88)	11.4 (10)	13.6 (12)	67.0 (24.7)	67.0 (25.1)	0.01 (11.7)
Group 3 (140)	24.3 (34)	27.1 (38)	70.3 (27.9)	72.4 (27.2)	2.1 (12.3)
Group 4 (37)	27.0 (10)	16.2 (6)	76.2 (24.2)	73.8 (25.4)	−2.3 (12.9)
Group 5 (34)	38.2 (13)	35.3 (12)	78.8 (28.6)	80.0 (27.6)	1.1 (6.7)
Group 6 (23)	60.9 (14)	65.2 (15)	87.2 (21.3)	85.0 (24.9)	−2.3 (10.6)
Group 7 (17)	58.8 (10)	70.6 (12)	88.3 (19.7)	87.7 (23.2)	−0.56 (10.7)
Total (462)	21.7 (100)	22.9 (106)	68.5 (28.1)	68.8 (28.3)	0.3 (12.4)

*Abbreviations:* BMI, Body mass index; QUALITY, Quebec Adipose and Lifestyle Investigation in Youth.

## Discussion

Recursive partitioning, a novel method in the study of neighborhoods and health, was used to examine how specific risk factors jointly influence obesity among children. Risk factors from different levels of influence based on a social ecological framework were considered. In this sample characterized by an overall high prevalence of familial obesity, successively higher BMI percentiles were found in children who cumulated individual, familial, and neighborhood environment risk factors. However, limited evidence for associations with 2-year changes in BMI percentile was found.

Classification trees are often unstable in the face of minor changes in the sample; using recursive partitioning in a different study sample is likely to yield a different classification tree. The relatively small data set used in this study further adds to the instability of the classification tree and yielded imprecise measures of associations, notably in the higher risk subgroups (e.g., n = 19 for group 7). Although findings may be difficult to reproduce and should be interpreted with caution, recursive partitioning allowed us to identify potentially highly obesogenic environments in the QUALITY study. Measures of associations reported in this study may be generalizable to Caucasian children with a parental history of obesity.

Recursive partitioning is a valuable data exploration method in the study of neighborhoods and health. It allows for the detection of higher order interactions within the data which would be challenging to examine using Generalized Linear Models. Other strengths of this study include the use of objective measures of obesity in children and both biological parents, PA, and neighborhood environment indicators, and the use of neighborhood definitions centered on each participant’s residential address.

It is well recognised in the literature that obesity is influenced by multiple risk factors stemming from multiple levels of influence, yet previous studies examined a limited range of risk factors simultaneously [4]. Recursive partitioning provides a unique method of analysis to generate hypotheses on how these multiple risk factors may jointly influence childhood obesity. In this analysis, individual and familial risk factors were selected first whereas neighborhood environment variables only emerged in latter branches of the classification tree. This may reflect strong associations between individual-level variables and obesity measured at the individual level but does not eschew the importance of contextual-level variables and obesity measured at both the individual and population levels [37,38]. Since obesity is likely the result of shared genetics, lifestyle and environmental risk factors, and since these relationships are difficult to disentangle in observational studies, contextual influences may be underestimated.

With respect to neighborhood characteristics, findings are consistent with the numerous studies that report more obesity among residents of socioeconomically disadvantaged neighborhoods [15]. At equal individual and familial risk and without consideration of subsequent splits, in this sample the prevalence of obesity was almost twice as high among children living in socioeconomically disadvantaged neighborhoods (52%) compared to those living in low disadvantage neighborhoods (28%). Among children living in socioeconomically disadvantaged neighborhoods, elements of the built and food environment, namely access to parks and convenience stores, further determined obesity. Findings suggest that neighborhood environment characteristics previously associated with childhood obesity (i.e., access to parks and convenience stores [6,11,13,39]) may be particularly influential for children who are already most vulnerable due to individual (i.e., physical inactivity) and familial risk factors (i.e., parental obesity).

Convincing evidence for associations between the classification tree subgroups and 2-year changes in BMI percentile was not found. Only children with ≥1 BMI-defined obese parents, not meeting PA guidelines, and with ≤1 abdominally obese parents showed an increase in BMI percentile at follow-up. This was the subgroup with the largest number of participants. Although other subgroups had coefficients of change of similar magnitude (i.e., Group 5), detection of associations may have been limited by the relatively small sample size. Selection bias may have resulted from the loss to follow-up of participants based on specific profiles of risk factors and on obesity. The duration of follow-up may have been insufficient to detect an effect on changes in BMI which typically occur slowly over time. Alternatively, determinants of obesity in cross-sectional associations may be different from those of obesity development which could explain why some cross-sectional findings are not reproduced in longitudinal analyses [40].

## Conclusion

Recursive partitioning allowed us to classify participants into qualitatively distinct subgroups based on a series of modifiable individual, familial and neighborhood environment risk factors. This provides some indications of potentially obesogenic environments and points to the “when, where, and for whom certain environmental attributes are most influential” on childhood obesity (p.101) [17]. Future studies in larger samples and with longer durations of follow-up are needed to better understand how different combinations of risk factors jointly predict obesity. Findings contribute to the growing body of evidence that supports the need for multi-level and multi-setting population approaches to obesity prevention [41]. In particular, interventions aimed at modifying neighborhood environments may be most beneficial for children who are already the most vulnerable due to individual and familial risk factors.

## Additional file

Additional file 1: Table S1-S3. Supplementary information.

## Abbreviations

BMI: body mass index
MVPA: moderate to vigorous physical activity
QUALITY: Quebec Adipose and Lifestyle Investigation in Youth

## References

[1] Wang Y, Lobstein T (2006). Worldwide trends in childhood overweight and obesity. Int J Pediatr Obes.

[2] Han JC, Lawlor DA, Kimm SY (2010). Childhood obesity. Lancet.

[3] Foresight (2007). Tackling obesities: future choices-project report.

[4] Birch LL, Davison KK (2001). Family environmental factors influencing the developing behavioral controls of food intake and childhood overweight. Pediatr Clin North Am.

[5] Sallis JF, Floyd MF, Rodriguez DA, Saelens BE (2012). Role of built environments in physical activity, obesity, and cardiovascular disease. Circulation.

[6] Galvez MP, Pearl M, Yen IH (2010). Childhood obesity and the built environment: a review of the literature from 2008–2009. Curr Opin Pediatr.

[7] Te Morenga L, Mallard S, Mann J (2013). Dietary sugars and body weight: systematic review and meta-analyses of randomised controlled trials and cohort studies. BMJ.

[8] Janssen I, Leblanc AG (2010). Systematic review of the health benefits of physical activity and fitness in school-aged children and youth. Int J Behav Nutr Phys Act.

[9] Silventoinen K, Rokholm B, Kaprio J, Sorensen TI (2010). The genetic and environmental influences on childhood obesity: a systematic review of twin and adoption studies. Int J Obes (Lond).

[10] Reilly JJ, Armstrong J, Dorosty AR, Emmett PM, Ness A, Rogers I (2005). Early life risk factors for obesity in childhood: cohort study. BMJ.

[11] Pate RR, O’Neill JR, Liese AD, Janz KF, Granberg EM, Colabianchi N (2013). Factors associated with development of excessive fatness in children and adolescents: a review of prospective studies. Obes Rev.

[12] Rahman T, Cushing RA, Jackson RJ (2011). Contributions of built environment to childhood obesity. Mt Sinai J Med.

[13] Dunton GF, Kaplan J, Wolch J, Jerrett M, Reynolds KD (2009). Physical environmental correlates of childhood obesity: a systematic review. Obes Rev.

[14] Safron M, Cislak A, Gaspar T, Luszczynska A (2011). Micro-environmental characteristics related to body weight, diet, and physical activity of children and adolescents: a systematic umbrella review. Int J Environ Health Res.

[15] Grow HM, Cook AJ, Arterburn DE, Saelens BE, Drewnowski A, Lozano P (2010). Child obesity associated with social disadvantage of children’s neighborhoods. Soc Sci Med.

[16] Carter MA, Dubois L (2010). Neighbourhoods and child adiposity: a critical appraisal of the literature. Health Place.

[17] Ding D, Gebel K (2012). Built environment, physical activity, and obesity: what have we learned from reviewing the literature?. Health Place.

[18] Diez Roux AV, Mair C (2010). Neighborhoods and health. Ann N Y Acad Sci.

[19] Strobl C, Malley J, Tutz G (2009). An introduction to recursive partitioning: rationale, application, and characteristics of classification and regression trees, bagging, and random forests. Psychol Methods.

[20] Breiman L, Friedman JH, Olshen RA, Stone CJ (1998). Classification and regression trees.

[21] Keegan TH, Hurley S, Goldberg D, Nelson DO, Reynolds P, Bernstein L (2012). The association between neighborhood characteristics and body size and physical activity in the California teachers study cohort. Am J Public Health.

[22] Lambert M, Van Hulst A, O’Loughlin J, Tremblay A, Barnett TA, Charron H (2012). Cohort profile: the Quebec adipose and lifestyle investigation in youth cohort. Int J Epidemiol.

[23] Marshall WA, Tanner JM (1969). Variations in pattern of pubertal changes in girls. Arch Dis Child.

[24] Marshall WA, Tanner JM (1970). Variations in the pattern of pubertal changes in boys. Arch Dis Child.

[25] Johnson RK, Driscoll P, Goran MI (1996). Comparison of multiple-pass 24-hour recall estimates of energy intake with total energy expenditure determined by doubly labeled water method in young children. J Am Diet Assoc.

[26] Canadian Nutrient File. [http://www.hc-sc.gc.ca/fn-an/nutrition/fiche-nutri-data/cnf_downloads-telechargement_fcen-eng.php].

[27] Troiano RP, Berrigan D, Dodd KW, Masse LC, Tilert T, McDowell M (2008). Physical activity in the United States measured by accelerometer. Med Sci Sports Exerc.

[28] Evenson KR (2008). Calibration of two objective measures of physical activity for children. J Sports Sci.

[29] Bastien M, Poirier P, Lemieux I, Després JP (2014). Overview of epidemiology and contribution of obesity to cardiovascular disease. Prog Cardiovasc Dis.

[30] Low income cut-offs after tax. [http://www.statcan.gc.ca/pub/75f0002m/2013002/tbl/tbl01-eng.htm]

[31] Van Hulst A, Gauvin L, Kestens Y, Barnett TA (2013). Neighborhood built and social environment characteristics: a multilevel analysis of associations with obesity among children and their parents. Int J Obes (Lond).

[32] Paquet C, Daniel M, Kestens Y, Leger K, Gauvin L (2008). Field validation of listings of food stores and commercial physical activity establishments from secondary data. Int J Behav Nutr Phys Act.

[33] Van Hulst A, Barnett TA, Gauvin L, Daniel M, Kestens Y, Bird M (2012). Associations between children’s diets and features of their residential and school neighbourhood food environments. Can J Public Health.

[34] Therneau T, Atkinson B, Ripley B (2013). rpart: Recursive partitioning. R package version 4.1-3.

[35] Zang H, Singer B (2010). Recursive partitioning and applications.

[36] An introduction to recursive partitioning using the RPART routines. [http://cran.r-project.org/web/packages/rpart/vignettes/longintro.pdf]

[37] Carroll-Scott A, Gilstad-Hayden K, Rosenthal L, Peters SM, McCaslin C, Joyce R (2013). Disentangling neighborhood contextual associations with child body mass index, diet, and physical activity: the role of built, socioeconomic, and social environments. Soc Sci Med.

[38] Jansen PW, Mensah FK, Nicholson JM, Wake M (2013). Family and neighbourhood socioeconomic inequalities in childhood trajectories of BMI and overweight: longitudinal study of Australian children. PLoS One.

[39] Leung CW, Laraia BA, Kelly M, Nickleach D, Adler NE, Kushi LH (2011). The influence of neighborhood food stores on change in young girls’ body mass index. Am J Prev Med.

[40] Timperio A, Jeffery RW, Crawford D, Roberts R, Giles-Corti B, Ball K (2010). Neighbourhood physical activity environments and adiposity in children and mothers: a three-year longitudinal study. Int J Behav Nutr Phys Act.

[41] Foltz JL, May AL, Belay B, Nihiser AJ, Dooyema CA, Blanck HM (2012). Population-level intervention strategies and examples for obesity prevention in children. Annu Rev Nutr.

