# Supplementary material for: Identifying risk profiles for childhood obesity using recursive partitioning based on individual, familial, and neighborhood environment factors
Source: Int J Behav Nutr Phys Act. 2015 Feb 15;12:17. doi: 10.1186/s12966-015-0175-7 (PMC4336734; doi:10.1186/s12966-015-0175-7)
Supplement: Additional file 1: Table S1-S3. — Supplementary information. [file 12966_2015_175_MOESM1_ESM.docx]

**SUPPLEMENTARY INFORMATION**

**Identifying risk profiles for childhood obesity using recursive partitioning based on individual, familial, and neighborhood environment factors**

**A) Definition of neighborhood prestige and neighborhood disadvantage**

Supplementary Table 1 presents results from a principal components analysis on census data. Rotated factor loadings and total variance in data explained by components are shown. Specifically, neighborhood disadvantage is defined by a high proportion of residents with income below the low income cut-offs, single parent families, unemployment, high mobility (more people who have lived ≤ 1 year at their current residence), and a low proportion of residents who own their home. The low income cut-offs (LICOs) are income thresholds below which a family will likely devote a larger share of its income on the necessities of food, shelter and clothing than the average family. The approach is essentially to estimate an income threshold at which families are expected to spend 20 percentage points more than the average family on food, shelter and clothing.^[[1]](#footnote-1)^ Neighborhood prestige is defined by a high proportion of residents who are university educated and more expensive residences.

**Table S1. Rotated factor loadings and total variance in data explained by components obtained through a principal component analyses for data from the QUALITY study in 2005-2008.**

|  | **Neighborhood variables** | **Rotated factor loadings** |
| --- | --- | --- |
| **2006 Census data** | **Component 1: Neighborhood disadvantage** |  |
|  | % households living below Statistics Canada’s low income cut-offs | 0.84 |
|  | % single parent families | 0.79 |
|  | % unemployment | 0.77 |
|  | % owner occupied residences | -0.93 |
|  | % who have moved in the past year | 0.65 |
|  | **Component 2: Neighborhood prestige** |  |
|  | % residents with university degree | 0.94 |
|  | Average value of owner occupied residences | 0.95 |
|  | **Total variance explained** | **72.6%** |

**B) Note on statistical analysis methods used to account for clustering of participants within schools**

Although recruitment for the QUALITY study was done within schools, there is relatively little clustering of participants (n=512) within schools (n=296). As shown in the two results tables below, estimates for associations obtained from generalized estimating equations (Model B) are similar to those obtained from multivariable linear regressions (Model A) both for associations at baseline (Table S2) and associations with 2-year changes in BMI (Table S3).

**Table S2.** Unadjusted and adjusted associations (beta coefficients and 95% CIs) between risk subgroups identified using recursive partitioning analysis and body mass index percentile among 512 QUALITY study participants at baseline (2005-2008) obtained from multivariable linear regression (Model A) and generalised estimating equation to adjust for the clustering of participants in schools (Model B^*^).

|  | **MODEL A** | **MODEL B** |
| --- | --- | --- |
|  | **Beta (95% CI)** | |
| Intercept | 78.4 (51.9; 105.0) | 78.5 (53.1; 103.8) |
| Group 1 (n=132), obesity prevalence 7.6% | Reference | Reference |
| Group 2 (n=97), obesity prevalence 11.3% | 12.3 (5.3; 19.3) | 12.3 (5.6; 18.9) |
| Group 3 (n=163), obesity prevalence 26.4% | 15.8 (9.6; 22.0) | 16.6 (10.0; 23.3) |
| Group 4 (n=39), obesity prevalence 28.2% | 22.6 (13.1; 32.1) | 23.0 (14.2; 31.9) |
| Group 5 (n=37), obesity prevalence 40.5% | 23.8 (14.1; 33.5) | 24.5 (14.3; 34.6) |
| Group 6 (n=25), obesity prevalence 60.0% | 31.8 (20.4; 43.1) | 32.9 (23.2; 42.6) |
| Group 7 (n=19), obesity prevalence 63.2% | 32.7 (19.9; 45.4) | 33.0 (22.0; 44.0) |
| Child’s age | -3.3 (-6.1; -0.5) | -3.5 (-6.2; -0.7) |
| Sex, boys (vs girls) | 6.7 (1.5; 11.8) | 7.5 (2.3; 12.8) |
| Puberty initiated at baseline (vs not initiated) | 10.4 (3.9; 16.9) | 11.2 (4.7; 17.6) |
| Parental education |  |  |
| ≥1 parent with university degree | Reference | Reference |
| ≥1 parent with technical/vocational/trade school degree | 5.0 (0.1; 9.9) | 5.5 (0.6; 10.3) |
| 2 parents with high school degree or less | 7.6 (-1.1; 16.2) | 7.7 (-1.3; 16.7) |

Abbreviations: CI, confidence interval; QUALITY, Quebec Adipose and Lifestyle Investigation in Youth

* GEE model with normal link function for 512 participants recruited in 296 schools

**Table S3.** Unadjusted and adjusted associations (beta coefficients and 95% CIs) between risk subgroups identified using recursive partitioning analysis and body mass index percentile among 462 QUALITY study participants at 2 year follow-up (2005-2011) obtained from multivariable linear regression (Model A) and generalised estimating equation to adjust for the clustering of participants in schools (Model B^*^).

|  | **MODEL A** | **MODEL B** |
| --- | --- | --- |
|  | **Beta (95% CI)** | |
| Intercept | 1.8 (-13.9; 17.5) | 0.005 (-13.3; 13.3) |
| Child’s BMI percentile at baseline | 0.90 (0.9; 0.9) | 0.90 (0.9; 0.9) |
| Group 1 (n=123), obesity prevalence 8.9% | Reference | Reference |
| Group 2 (n=88), obesity prevalence 13.6% | 1.5 (-1.8; 4.9) | 1.5 (-2.0; 5.0) |
| Group 3 (n=140), obesity prevalence 27.1% | 3.6 (0.5; 6.6) | 3.8 (0.4; 7.2) |
| Group 4 (n=37), obesity prevalence 16.2% | -0.1 (-4.7; 4.4) | -0.2 (-5.1; 4.6) |
| Group 5 (n=34), obesity prevalence 35.3% | 3.8 (-1.0; 8.6) | 3.4 (-0.1; 6.8) |
| Group 6 (n=23), obesity prevalence 65.2% | 1.0 (-4.6; 6.6) | -0.4 (-5.3; 4.5) |
| Group 7 (n=17), obesity prevalence 70.6% | 2.5 (-3.8; 8.8) | 2.6 (-3.3; 8.6) |
| Child’s age at follow-up, years | 0.1 (-1.3; 1.5) | 0.5 (-0.8; 1.8) |
| Sex, boys (vs girls) | 0.7 (-1.8; 3.1) | -0.07 (-2.6; 2.5) |
| Puberty initiated at follow-up (vs not initiated) | 2.6 (-0.3; 5.5) | 0.9 (-1.7; 3.5) |
| Parental education |  |  |
| ≥1 parent with university degree (reference) | Reference | Reference |
| ≥1 parent with technical/vocational/trade school degree | -0.03 (-2.4; 2.3) | -0.2 (-2.5; 2.2) |
| 2 parents with high school degree or less | 0.5 (-3.8; 4.8) | 0.4 (-3.9; 4.6) |

Abbreviations: BMI, body mass index; CI, confidence interval; QUALITY, Quebec Adipose and Lifestyle Investigation in Youth

* GEE model with normal link function for 462 participants recruited in 296 schools

1. Statistics Canada. Low income cut-offs; 2012 [cited 2013 Apr 12]. Available from: <http://www.statcan.gc.ca/pub/75f0002m/2012002/lico-sfr-eng.htm> [↑](#footnote-ref-1)
